# 2168. Viral DNAemia and Herpesvirus Seropositivity are Associated with Mortality in Pediatric Patients with Severe Sepsis

**DOI:** 10.1093/ofid/ofac492.1788

**Published:** 2022-12-15

**Authors:** Stephanie S Cabler, Gregory Storch, Jason B Weinberg, Andrew H Walton, Karen Brengel-Pesce, Zachary Aldewereld, Russell K Banks, Joseph Carcillo

**Affiliations:** Washington University in St. Louis, St. Louis, Missouri; Washington University in St. Louis, St. Louis, Missouri; University of Michigan, Ann Arbor, Michigan; Washington University in St. Louis, School of Medicine, St. Louis, Missouri; Biomerieux, Lyon, Rhone-Alpes, France; University of Pittsburgh Medical Center, Pittsburgh, Pennsylvania; The University of Utah, Salt Lake City, Utah; University of Pittsburgh Medical Center, Pittsburgh, Pennsylvania

## Abstract

**Background:**

Sepsis is a leading cause of pediatric mortality. Little attention has been paid to latent and reactivated viral infection in critically ill children with sepsis. We report a large multi-center study of viral DNAemia and herpesvirus seropositivity in pediatric patients with severe sepsis.

**Methods:**

We enrolled 401 pediatric patients from 9 Pediatric Intensive Care Units (PICUs). Patient samples were tested via qPCR for EBV, CMV, HSV, adenovirus, HHV6, BK and parvovirus B19 DNA. CMV, EBV, HSV and HHV6 IgG were also measured to classify patients as having no infection (IgG-negative without DNAemia), acute infection (IgG-negative with DNAemia), reactivated infection (IgG-positive with DNAemia), or latent infection without reactivation (IgG-positive without DNAemia).

**Results:**

55% of enrolled patients were male, 39% previously healthy, and 27% immunocompromised. 56% had documented infection(s) on enrollment (63% bacterial, 50% viral, and 2% fungal). 11% died in the PICU.

Viral DNAemia was detected in 61% of immunocompromised patients and 46% of non-immunocompromised patients. DNAemia with 2 or more viruses on study, detected in 21% of patients, was independently associated with increased mortality in both immunocompromised (OR 5.5 [1.6, 22.6] p=0.023) and non-immunocompromised patients (OR 3.9 [1.4, 11.5] p=0.015). Viral detection was due to reactivated infection in 91% of patients with EBV DNAemia, 63% with CMV, and 100% with HSV and HHV-6, making acute infection rare.

Viral seropositivity (HSV 33%, CMV 42%, EBV 61%, HHV6 98%) and latent infection (HSV 30%, CMV 35%, EBV 45%, HHV6 75%) were both common. Adjusted mortality was higher in patients seropositive for EBV (OR 9.1 [2.8, 172.8], p=0.002) compared to those seronegative for EBV. For HHV-6, mortality was higher in patients with viral reactivation compared to latent infection (18% vs. 7%, p=0.024). Mortality in patients with no detected viral infection (IgG-negative without DNAemia) was low (≤ 5% for each virus).

Pediatric Sepsis Mortality and Viral DNAemia

**Figure:**
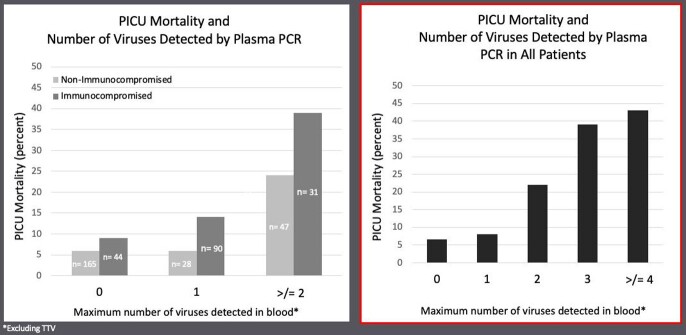

ICU mortality was significantly associated with the number of viruses detected during ICU stay in both non-immunocompromised patients (light grey bars) and immunocompromised patients (dark grey bars). In patients with 0 viruses detected, morality was less than 10% in both groups. If 1 virus was detected, mortality was 6% in non-immunocompromised patients and 14% in immunocompromised patients. In the 47 non-immunocompromised patients in which 2 or more viruses were detected, mortality was almost 25% and in the 31 immunocompromised patients mortality neared 40%.

Among all patients, additional viral detection of 3, 4 or more viruses by PCR continued to increase risk of death, with mortality nearing 45% in patients who had 4 or more viruses detected during their ICU admission.

**Conclusion:**

Viral DNAemia was common and associated with mortality in pediatric patients with severe sepsis. DNAemia with 2 or more viruses increased mortality risk, even in previously healthy patients. EBV seropositivity was strongly associated with PICU mortality, independent of concurrent viral DNAemia.

**Disclosures:**

**Andrew H. Walton, MS**, RevImmune: Grant/Research Support|Thermo Fisher: Spouse employed by Thermo Fisher.

